# Extremophilic Solutions: The Role of Deinoxanthin in Counteracting UV-Induced Skin Harm

**DOI:** 10.3390/cimb45100528

**Published:** 2023-10-16

**Authors:** Mehmet Kuzucu

**Affiliations:** Department of Biology, Faculty of Arts and Sciences, Erzincan Binali Yildirim University, Erzincan 24100, Türkiye; mkuzucu@erzincan.edu.tr

**Keywords:** deinoxanthin, UV-induced damage, melanogenesis, *Deinococcus radiodurans*

## Abstract

This research delved into the protective capacities of deinoxanthin, a carotenoid present in Deinococcus radiodurans, against UVA- and UVB-mediated skin damage using human fibroblast foreskin cells (HFF-1). Using the MTT assay, HFF-1 cells treated with 10 µM DNX displayed 20% and 31.7% higher viability than the positive (Vitamin C-treated) and negative (DNX-untreated) control groups, respectively, upon 100 mJ/cm^2^ UVB exposure. At 24 J/cm^2^ UVA, 20 µM DNX-treated cells showed 80.6% viability, exceeding the positive and negative control groups by 28.6% and 33.6%, respectively. Flow cytometry analysis revealed that cells treated with DNX and exposed to 24 J/cm^2^ UVA exhibited a 69.32% reduction in apoptotic processes compared to untreated cells. Similarly, when exposed to 100 mJ/cm^2^ UVB, DNX-treated cells demonstrated a 72.35% decrease in apoptotic processes relative to their untreated counterparts. DNX also displayed dose-dependent inhibition on tyrosinase activity. The study emphasized DNX’s antioxidative capacity, evident in its modulation of superoxide dismutase activity and measurements of Malondialdehyde and intracellular reactive oxygen species levels. DNX-treated cells exhibited higher hydroxyproline levels, suggesting healthier collagen production. Additionally, the wound-healing assay method confirmed an accelerated healing rate in DNX-treated cells. Conclusively, DNX offers significant protection against UV-induced skin damage, emphasizing its potential for skincare and therapeutics.

## 1. Introduction

According to the Lambda-CDM concordance model, our universe, which is estimated to be 13.798 ± 0.037 billion (13.798 ± 0.037 × 10^9^) years old, has witnessed many extraordinary atmospheric and climatic changes over time [1].

Since the dawn of the universe, life has been challenged under various demanding physical and chemical conditions such as high temperatures, high pressures, low water quantities, fluctuating atmospheric compositions, and high radiation. Under these challenging conditions, organisms that managed to persist have passed on their survival experiences to subsequent generations through their genetic structures, offering us insights into their resilience.

In certain environments, specific organisms have evolved to withstand incredibly harsh conditions. Known as extremophiles, these organisms not only survive but also produce various natural bioactive compounds. Among these compounds, carotenoids are especially noteworthy [2]. Carotenoids act as precursors for some vitamins, which are crucial for physiological processes including cell differentiation and growth, etc. [3], and as antioxidants as they help alleviate the damage prompted by oxidation and high sun exposure on cells and, hence, can be used in cosmetics and pharmaceuticals [4]. Moreover, carotenoids have DNA repair capabilities and they can repair undesired cellular functions. Carotenoids have also been demonstrated to have a powerful effect on protecting skin cells from damage induced by ultraviolet radiation [5,6,7].

*Deinococcus radiodurans* is a red-pigmented bacterium well known for its extreme resistance to oxidizing agents, DNA damage, ultraviolet radiation, and γ-rays [8]. Deinoxanthin (DNX), a main carotenoid in *D. radiodurans*, gives the bacterium its characteristic features such as the DNA repair capability and powerful enzymatic/non-enzymatic antioxidant defense system [9,10]. DNX also provides proteome protection, which is important for survival after radiation since protein activity is required for necessary processes including transcription, translation, and the repair of DNA [8,9]. Farci et al. [9] demonstrated that especially under desiccation, DR_2577, an S-layer protein, binds deinoxanthin, and so protects *D. radiodurans* from UV radiation. Chen et al. investigated the role of *D. radiodurans*, an extremophile, in allergic diseases, focusing on its cellular components. Their findings revealed that among the cellular components of *D. radiodurans*, only the cell wall (DeinoWall) suppressed Th2 cytokine production and prevented atopic dermatitis in mice. The study further demonstrated that DeinoWall promotes the maturation of dendritic cells, favoring Th1-biased immunity, which may regulate allergic reactions. Overall, DeinoWall might offer a potential therapeutic avenue for allergic diseases [11]. However, the role of deinoxanthin and its mechanisms in UV radiation-caused skin damage is still not defined.

The epithelial tissue is essential in protecting organisms against various environmental adversities by forming a nearly uninterrupted intercellular layer. This layer acts as a defense mechanism against physical, chemical, and microbial threats, as well as allergens. Notably, the characteristics of this protective layer vary depending on its location, such as the skin, digestive system, and respiratory pathways. The proliferation of environmental pollutants post the industrial revolution, including particulate matter, microplastics, exhaust gases, ozone, and the increased toxins from cleaning agents, detergents, and surfactants, has heightened health concerns [12].

Furthermore, impairments in the epithelial layer, sometimes termed ‘leaky epithelium’, have been identified in organs affected by diseases like asthma, chronic rhinosinusitis, allergic rhinitis, atopic dermatitis, eosinophilic esophagitis, and digestive disorders such as celiac and inflammatory bowel diseases. Such observations have led to the hypothesis highlighting the pivotal role of the epithelial barrier in the onset and progression of these diseases [13,14].

Naturally occurring UV radiation is one of the most detrimental environmental agents for the skin, leading to photocarcinogenesis [10], photosensitivity disorders [15], and photoaging [16]. UV radiation can be classified into three primary types: UVA, UVB, and UVC. Among these, UVC (200–280 nm) is mainly absorbed by the ozone layer and does not typically reach the earth’s surface [17]. On the other hand, UVB radiation (290–320 nm) accounts for about 5% of total solar UV radiation and UVA (320–400 nm) the remaining 95%, both of which are very harmful to the skin [18]. Depending on the wavelength of the radiation, UV demonstrates detrimental effects on cells in different ways by directly or indirectly acting on DNA [19]. Actually, UVB is accepted as being more mutagenic and carcinogenic than UVA because of absorbing photons at this wavelength directly by DNA bases [19,20]. Under exposure to both UVA and UVB radiation on the skin, oxidative damage is triggered in keratinocytes, resulting in the formation of reactive nitrogen species (RNS) and reactive oxygen species (ROS) which react with proteins and lipids. Further, excessive ROS causes the activation of enzymes, such as hyaluronidase, matrix metalloproteinases (MMPs), and elastase, which derange collagenous and elastic fibers and eventually result in the impairment of the extracellular matrix and the structural frame of skin [21]. Meanwhile, ROS and RSN activate the expression of tyrosinase, which eventually promotes the overproduction of melanin [22]. Although melanin has a crucial role in protecting the skin, its abnormal production leads to a large number of serious dermatological disorders including melasma, post-inflammatory hyperpigmentation, and skin cancer [23]. Hence, the controlling of melanin production via regulation of the gene for tyrosinase, the rate-limiting enzyme in melanin synthesis, is important to protect the skin from damage by UV photons [24].

Artificial sunscreens have recently been used to protect the skin from the harmful effects of UV radiation mentioned above [25]. However, conventional UV filters are highly debated due to their photostability and environmental effects [26,27]. Nowadays, near attention has been paid to carotenoids because of their amicable safety profile and potentially valuable biological activities, and exploring carotenoids with anti-oxidant, anti-inflammatory, and humectant features for the prevention and/or treatment of skin UV radiation damage has become a new hot spot in skin care research [28]. For this reason, the current report firstly investigates the biological activity of deinoxanthin extract from *D. radiodurans* against skin damage caused by UVA and UVB radiations in an in vitro model using human fibroblast foreskin cell line HFF-1, with a special focus on the direct relationship of deinoxanthin with oxidative stress and melanin synthesis.

## 2. Materials and Methods

### 2.1. Chemicals and Reagents

The following reagents were obtained from Sigma-Aldrich (St. Louis, MO, USA): 3-(4,5-Dimethyl-2-thiazolyl)-2,5-diphenyl-2H-tetrazolium bromide (MTT), Dimethyl sulfoxide (DMSO), vitamin C, L-3,4-dihydroxyphenylalanine (L-DOPA), Triton X-100, nitro blue tetrazolium (NBT) riboflavin, ethylenediaminetetraacetic acid (EDTA), thiobarbituric acid (TBA), trichloroacetic acid (TCA), 2′,7′-dichlorofluorescin diacetate (DCFH-DA).

The Muse^®^ Annexin V & Dead Cell Kit was obtained from Luminex (Luminex Corporate, Austin, TX, USA). Additionally, Dulbecco’s Modified Eagle’s Medium (DMEM), Fetal Bovine Serum (FBS), Penicillin-Streptomycin solution, Trypsin-EDTA solution, L-glutamine, and Dulbecco’s Phosphate Buffered Saline (DPBS) were obtained from Biological Industries, Kibbutz Beit Haemek, Israel. DNX was provided from a purified (Figure S1) stock produced by Seda KILINÇ in a joint venture between Erzincan Binali Yıldırım University and Marmara University [29].

### 2.2. Cell Culture

The human foreskin fibroblast cell line HFF-1 was obtained from ATCC (American Type Culture Collection, Manassas, VA, USA, LGC Promochem, Teddington, UK). HFF-1 cells were cultured in DMEM supplemented with 10% FBS and 1% penicillin/streptomycin. The cells were grown at 37 °C in a sterilized 5% CO_2_ atmosphere by filters. DNX and vitamin C (VitC) were dissolved in DMSO and DMEM, respectively. Cell culture experiments were carried out within a biosafety cabinet (NUVE, Istanbul, Turkey).

### 2.3. Cytotoxicity Assay

The MTT method was used to assess the cytotoxicity of the DNX against HFF-1 cells [30]. Initially, the cells were incubated in high-glucose DMEM medium containing 15% fetal bovine serum at 37 °C under 90% humidity and 5% CO_2_ until they reached 90% confluence. Subsequently, 5 × 10^3^ cells were plated into individual wells of a 96-well plate, followed by a 24 h incubation under consistent conditions to ensure cell attachment. The medium was replaced with DNX solutions of predetermined concentrations, containing less than 1% DMSO, prepared in DMEM (0, 5, 10, 20, 40, 80, 160, and 320 µM). Following a 24 and 48 h incubation period under consistent conditions, the medium with DNX was substituted with 90 µL of DMEM and a mixture of 10 µL of MTT (5 mg/mL) in DPBS (pH 7.4). Thereafter, another 5 h incubation ensued under identical conditions. Post incubation, the MTT-containing medium was discarded, and wells received 100 µL of DMSO. The absorbance for each well was then measured at 570 nm using a microplate reader from BioTek, Burlington, VT, USA. While Methotrexate functioned as the positive control, the negative control wells were deemed to represent 100% viability. The half-maximal inhibitory concentration (IC50) of DNX at which 50% of the cells died vs. the concentration was calculated using a variable slope (four parameters) nonlinear regression curve according to Equation (1).

(1) Y=Min+(Max−Min)1+XIC50Hill coefficient

“Y” represents the absorbance observed at 570 nm in cells exposed to various concentrations of DNX. The terms “Min” and “Max” depict the absorbance of cells that have not been treated with DNX and cells treated with the highest concentration of DNX, respectively. “X” signifies the concentration of DNX. The Hill coefficient provides insights into the nature of the dose–response relationship. Values greater than 1 suggest positive cooperativity, while those less than 1 indicate negative cooperativity. A coefficient of 1 indicates a standard dose–response curve without cooperativity.

### 2.4. Assessing Deinoxanthin’s Protective Role in HFF-1 Cells Subjected to UVA and UVB Radiation

#### 2.4.1. Assessment of Cell Viability

The research conducted experiments on groups exposed to specified concentrations of DNX and VitC (200 µM), established from cytotoxicity test outcomes. In the present investigation, VitC was incorporated as it is a water-soluble micronutrient commonly employed as a benchmark in research focusing on the attenuation of ultraviolet-induced cellular damage [31,32,33,34,35]. In addition, a negative control group (non-irradiated) and a positive control group (exposed to UVA doses of 8, 16, and 24 J/cm^2^ and UVB doses of 35, 70, and 100 mJ/cm^2^) were also included. Initially, 100 µL of growth medium-containing cells (5 × 10^3^) were seeded in 96-well plates, followed by a 24 h incubation period. The cells were treated with either various concentrations of DNX (0, 5, 10, and 20 µM) or 200 µM VitC in FBS-free DMEM for 12 h. After discarding the supernatant, each well underwent three washes with 150 µL of DPBS (pH 7.4). Subsequently, 200 µL of DPBS were added to each well, followed by UVA or UVB irradiation using the photoreactor system (LZC-4X, Luzchem, Ottawa, ON, Canada) consisting of UVA lamps (2.25 mW/cm^2^) and UVB lamps (0.8 mW/cm^2^). The lamp’s intensity was gauged utilizing an HD 2302.0 radiometer (Delta OHM, Padova, Italy) equipped with both UVA and UVB sensors. Post irradiation, the DPBS was discarded and replaced with 100 µL of DMEM without FBS, which was then incubated for 12 h. Subsequently, cell viability was determined via the MTT assay, as outlined earlier in the cytotoxicity test.

#### 2.4.2. Apoptosis Assay

The protective properties of DNX from the apoptotic effects of UVB and UVA on the human foreskin fibroblast cells were studied using an Annexin V-FITC/PI double-staining assay. To conduct the assay, 2 × 10^5^ cells were seeded in a 6-well culture plate and treated with various concentrations (0, 5, 10, and 20 µM) of DNX and VitC (200 µM) in FBS-free DMEM for 12 h. After discarding the supernatant, each well was thoroughly rinsed with DPBS (pH 7.4) on three separate occasions. Subsequently, 2 mL of PBS was added to each well, after which the cells underwent irradiation at intensities of 24 J/cm^2^ for UVA and 100 mJ/cm^2^ for UVB. Post irradiation, the DPBS was carefully aspirated, and the cells were supplied with 2 mL of DMEM devoid of FBS, followed by a 12 h incubation period. Afterward, the apoptotic effects of UVA and UVB exposure were evaluated as per the instructions provided by the manufacturer (Muse^®^ Annexin V & Dead Cell Kit, Merck Millipore, Warsaw, Poland).

#### 2.4.3. Determination of Tyrosinase Activity

HFF-1 cells were cultured and seeded in 96-well plates at a density of 5 × 10^3^ cells per well and allowed to adhere overnight. Prior to UV exposure, the cells were treated with varying concentrations of DNX (0, 5, 10, and 20 µM) and VitC (200 µM) for 12 h. Tyrosinase activity was assessed using a colorimetric assay based on L-DOPA oxidation [36]. After the post-UV incubation period, the cells were washed with DPBS and lysed in 50 mM sodium phosphate buffer (pH 6.8) containing 1% Triton X-100. The cell lysates were centrifuged at 12,000× *g* for 15 min at 4 °C, and the supernatants were collected. In a 96-well plate, 80 µL of supernatant was mixed with 20 µL of 5 mM L-DOPA in sodium phosphate buffer (pH 6.8). The reaction mixture was incubated for 1 h at 37 °C. Tyrosinase activity was determined by measuring the absorbance at 475 nm with 700 nm as reference using a microplate reader. The activity was expressed as a percentage of the control group (untreated, non-irradiated cells).

#### 2.4.4. Determination of Superoxide Dismutase Activity

To assess the protective effects of DNX and VitC on cells exposed to UVA (24 J/cm^2^) and UVB (100 mJ/cm^2^) irradiation, an SOD assay based on the McCord and Fridovich method was performed [37]. The experimental protocol is as follows: Cells were seeded in a 6-well culture plate at a density of 2 × 10^5^ cells per well. They were then treated with various concentrations of DNX (0, 5, 10, and 20 µM) and VitC (200 µM) in FBS-free DMEM and incubated for 12 h. After the incubation period, the supernatant was removed, and each well was washed three times with DPBS at pH 7.4. Next, 2 mL of PBS was added to each well, and the cells were subjected to the specified doses of both UVB and UVA irradiation.

Following irradiation, DPBS was carefully removed, and each well was supplemented with 2 mL of DMEM devoid of FBS. Thereafter, a further 12 h incubation period was undertaken for the cells. After this incubation, the cell lysates were collected and prepared for the SOD assay. For the SOD assay, a reaction mixture containing 50 mM phosphate buffer (pH 7.8), 0.1 mM EDTA, 50 µM NBT, and 10 µM riboflavin was prepared. An appropriate amount of cell lysate was added to the reaction mixture, ensuring that the total volume was consistent across all samples. The samples were then illuminated with a fluorescent light source for 10–15 min at room temperature to initiate the photochemical reaction. The absorbance of the samples at 560 nm was measured using a spectrophotometer, and the SOD activity was calculated by comparing the inhibition of the NBT reduction in the presence of the cell lysates with a blank control containing all reagents except for the cell lysate. Data were expressed as unit of SOD per mg of protein.

#### 2.4.5. Measuring Malondialdehyde Levels

Lipid peroxidation was evaluated by measuring Malondialdehyde (MDA) levels using the TBA assay [38]. HFF-1 cells (2 × 10^5^) were homogenized on ice in buffer and lysates were prepared. Aliquots of each lysate were mixed with 10% TCA and 1% TBA, and the mixture was heated at 90 °C for 10 min to form a TBA-MDA complex. Upon halting the reaction with an ice bath, the samples underwent centrifugation at 10,000× *g* for a duration of 5 min. The absorbance of the supernatant was recorded at 532 nm. The non-specific absorbance at 600 nm was then deducted, and the MDA concentration was calculated (from three replicate measurements per treatment) using an extinction coefficient of 0.155 µmol L^−1^cm^−1^. MDA levels were calculated from a standard curve generated using known concentrations of MDA (1,3,3-tetramethoxypropane) and normalized to the total protein content of each sample. Results are expressed as MDA levels in nmol per mg of total protein.

#### 2.4.6. Hydroxyproline Measurement

After the recovery period following ultraviolet radiation exposure and treatment with DNX and VitC, HFF-1 cells were collected and washed with PBS. Cell pellets were obtained by centrifugation at 300× *g* for 5 min at 4 °C. The cell pellets were then hydrolyzed in 6 M HCl at 110 °C for 24 h to release hydroxyproline from the intracellular collagen. After hydrolysis, the samples were centrifuged at 12,000× *g* for 10 min, and the supernatants were collected for hydroxyproline measurement. The hydroxyproline content in the cell lysates was measured using Abcam’s Hydroxyproline Assay Kit (Colorimetric) (Product code: ab222941) according to the manufacturer’s instructions. The absorbance was measured at 560 nm using a microplate reader. The hydroxyproline content in the samples was calculated based on the standard curve, and the results were normalized to the protein content in the cell lysates, determined by the Bradford protein assay. Data were expressed as micrograms of hydroxyproline per mg of protein.

#### 2.4.7. Intracellular Reactive Oxygen Species Quantification

HFF-1 cells were cultured and seeded in 24-well plates at a density of 2 × 10^5^ cells per well and allowed to adhere overnight. Prior to UV exposure, the cells were treated with varying concentrations of DNX and VitC (200 µM) for 12 h. To measure the intracellular reactive oxygen species (ROS) levels, the DCFH-DA probe was utilized, as described by Zhang et al. [39]. After the post-UV incubation period, the cells were washed with DPBS and incubated with 10 µM DCFH-DA in serum-free DMEM for 30 min at 37 °C in the dark. Subsequently, the DCFH-DA-containing medium was removed, and the cells were washed three times with PBS. Fluorescence intensity was measured using RF-5301PC (Shimadzu Corp., Kyoto, Japan) with excitation and emission wavelengths set at 485 nm and 535 nm, respectively. Protein quantification was performed using Bradford method and it was used to normalize the results.

#### 2.4.8. Real-Time Monitoring and Assessment of Wound Healing Progress

Cells were seeded in 6-well plates at a concentration of 5 × 10^5^ cells/well and cultured until they achieved a confluent monolayer. Using a sterile 10-μL pipette tip, uniform scratches were created across the monolayer. Afterward, the plates were gently rinsed with Dulbecco’s phosphate-buffered saline to remove any detached cell debris. Subsequent to this, specific groups of cells were treated with either DNX or VitC, while a control group remained untreated. Following a 2 h incubation, all groups, excluding the control, were subjected to UVA (24 J/cm^2^) and UVB (100 mJ/cm^2^) radiation. Observations of the wound healing process were systematically made using an inverted microscope (Nikon, Eclipse Ti2-U, 10× objective) at specified intervals: 0, 6, 12, and 24 h post scratching. To assess cell migration, the percentage of the remaining cell-free region in comparison to the original scratched area was calculated. This study’s findings were based on data aggregated from at least three independent experiments. For a more detailed analysis of the wound gap, the Image-Pro Plus 6.0 Software was employed. The rate of wound closure was ascertained using Equation (2).

(2) $$Wound\;Closure\;(\%)=\left(\left(W_{0}\right.\right.-\left.W_{t}\right)/\left.W_{0}\right)\times 100$$

$W_{0}$ and $W_{t}$ represent the wound sizes at hours 0 and t, respectively.

### 2.5. Statistical Analysis

Data analysis was conducted employing a one-way ANOVA using SigmaPlot 14 (Systat Software, Inc., San Jose, CA, USA). Subsequent comparisons were made with Tukey’s multiple-range test. Results are displayed as mean values accompanied by the standard deviation (SD). A *p*-value less than 0.05 was considered to denote statistical significance.

## 3. Results

### 3.1. Cytotoxicity Assay

**Figure 1:**
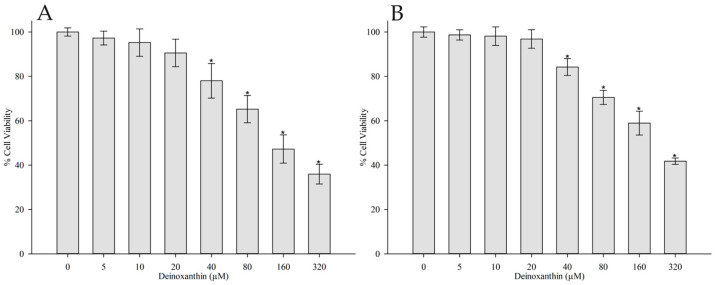
Effects of varying DNX concentrations on the cytotoxicity of human foreskin fibroblast cells (HFF-1) observed at (**A**) 24 h and (**B**) 48 h. Values are represented as mean ± standard error of mean. * *p* < 0.05 vs. 0 µM DNX groups.

To establish appropriate non-cytotoxic concentrations of DNX for further experiments, HFF-1 cells were treated with a range of DNX concentrations (0, 5, 10, 20, 40, 80, 160, and 320 µM) for 24 and 48 h. Cell viability was assessed using the MTT assay. Based on the MTT assay results, IC50 values of DNX on HFF-1 cells were found to be 95.0891 µM and 117.3288 µM for 24 h and 48 h, respectively (Figure 1). According to the results obtained, cell viability percentages of 5, 10, and 20 M concentrations of DNX in 24 and 48 h incubations are between 90.5 and 97.2% and 96.8 and 98.6%, respectively. This identified a safe dosage range for DNX when applied to the HFF-1 cell line, with cell viability exceeding 90%, signifying its safety. This selected dosage range was then used for subsequent investigations. Therefore, subsequent experiments were conducted using 5, 10, and 20 µM concentrations of DNX, which were determined to be non-cytotoxic and suitable for evaluating the potential protective effects of DNX against UV-induced damage in HFF-1 cells.

### 3.2. Evaluating the Protective Effects of Deinoxanthin on HFF-1 Cells Exposed to UVA and UVB Radiation

#### 3.2.1. Cell Viability Assay

**Figure 2:**
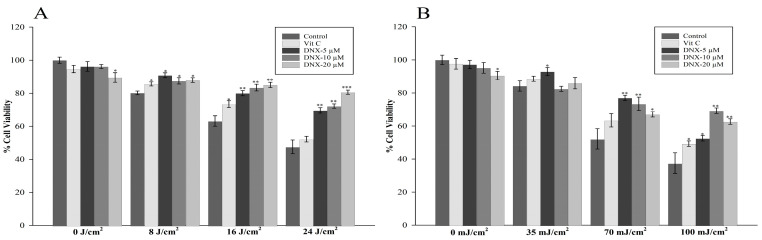
Protective effects of varying concentrations of DNX on HFF-1 cells exposed to UVB and UVA radiation observed at (**A**) UVA and (**B**) UVB. Statistical significance in this figure indicates the observed differences between the control group and the groups treated with varying concentrations of DNX and VitC, all subjected to the same UV dosage levels. The results are presented as the mean ± s.e.m. (*n* = 6). * *p* < 0.05; ** *p* < 0.001; *** *p* < 0.005.

The MTT assay was utilized to assess the protective roles of distinct DNX concentrations (0, 5, 10, and 20 µM) on HFF-1 cells under UVB and UVA irradiation. Relative to non-irradiated cells, HFF-1 cell viability declined to 84.2% following irradiation at 35 mJ/cm^2^ UVB (Figure 2B). A further reduction in viability was noted at 70 mJ/cm^2^ UVB, with cells displaying only 52.2% viability. When exposed to a UVB irradiation intensity of 100 mJ/cm^2^, cell viability drastically decreased to 37.7%.

In the non-exposed group, it was ascertained that 20 µM DNX demonstrated a cytotoxic effect by reducing cell viability by approximately 9.5%. Among the cells irradiated with 35 mJ/cm^2^ UVB, those treated with 5 µM DNX exhibited the highest viability at 92.9%, relative to the control. When analyzing the UVB-irradiated dose-specific groups, the most significant statistical difference was observed in the cells treated with 10 µM DNX at the 100 mJ/cm^2^ dose level. These cells showed a 31.7% higher viability rate compared to the negative control group, and a 20% increase when juxtaposed with the group treated with VitC at the same concentration.

In experiments involving UVB radiation at an intensity of 100 mJ/cm^2^, all tested concentrations of DNX outperformed VitC in terms of cellular protection. For subsequent investigations, a concentration of 10 µM DNX was selected for further evaluation against UVB exposure at 100 mJ/cm^2^. This decision was informed by observations that cells exposed to 10 µM DNX demonstrated an average viability rate that was 10.5% greater than those treated with a 20 µM concentration under the same UVB conditions.

In the experimental setup, the impact of pre-treating with DNX was assessed by contrasting each dosage cohort with a corresponding control group across UVA exposures of 0, 8, 16, and 24 J/cm^2^ (Figure 2A). Additionally, the effectiveness of VitC was appraised in the same context. In the experimental conditions involving 8 J/cm^2^ UVA exposure, the peak cell viability was recorded at approximately 91% following the administration of 5 µM DNX. In the cohort exposed to 16 J/cm^2^ UVA radiation, the control group exhibited 63.2% viability, whereas the highest level of cell viability was observed in the DNX-treated cells at 85%, utilizing a 20 µM concentration. Furthermore, VitC displayed diminished cell viability at 73.4%, when compared to DNX under the same UVA exposure. During the 24 J/cm^2^ UVA irradiation, the cell viability metrics were as follows: 47% in the control group, around 52% with VitC treatment, and an elevated 80.6% in HFF-1 cells treated with 20 µM DNX.

#### 3.2.2. Apoptosis Assay

**Figure 3:**
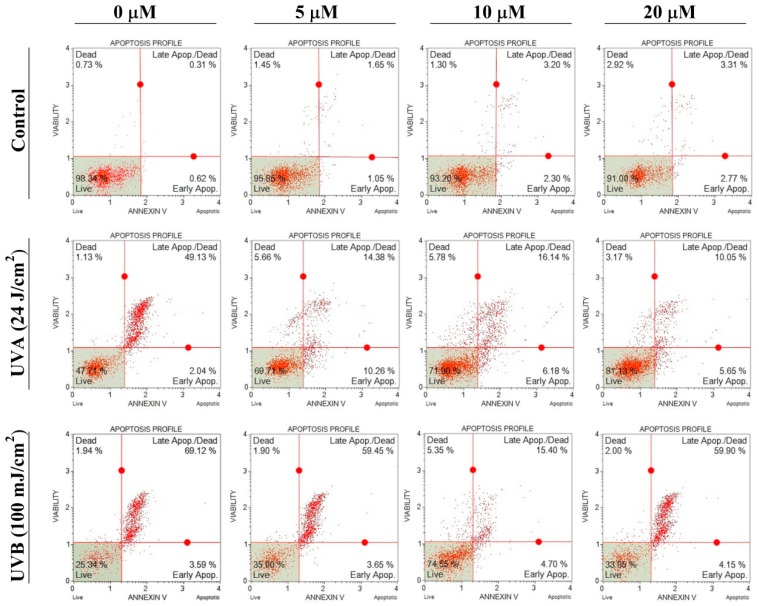
Evaluation of apoptosis-associated parameters in HFF-1 cells: viable and apoptotic cells were quantified. Each group used three wells for Annexin V-FITC/PI staining. Population analyses were performed in triplicate, and the entire assay was repeated three times.

To evaluate the anti-apoptotic efficacy, the maximal selected doses of UVA and UVB (24 J/cm^2^ and 100 mJ/cm^2^, respectively) were employed. This approach aimed to ascertain the uppermost protective capacity of DNX within the chosen concentration spectrum. In this study, the protective efficacy of DNX against UVA and UVB-induced apoptosis in HFF-1 cells was assessed using the Annexin V-FITC/PI double-staining assay. Cells were treated with varying DNX concentrations, exposed to specified UVA and UVB intensities, and subsequent apoptotic effects were quantified post-irradiation using the Muse^®^ Annexin V & Dead Cell Kit (Figure 3).

From the experimental outcomes, the viability of cells, initially at 98.34% in the control, witnessed a substantial decline under the influence of DNX, echoing the findings from the MTT analysis. In the control, the minimal cell viability was recorded at a robust 91% with 20 µM DNX. The apex of viability for HFF-1 cells exposed to 24 J/cm^2^ UVA was identified as 81.13% in the cohort pre-treated with 20 µM DNX. This represents a 33.42% elevation in viability compared to the control. The striking congruence between this result and the MTT findings underscores the internal consistency of the investigation.

In the exposure to 100 mJ/cm^2^ UVB, the HFF-1 cells demonstrated a peak viability of 74.55% when pre-treated with 10 µM DNX. In contrast, in the absence of DNX treatment, cell viability stood at 25.34%, with 3.59% of cells in the early apoptotic phase and 69.12% in the late apoptotic phase. A notable observation was the reduced cell viability at 20 µM DNX under UVB exposure compared to UVA exposure. This might be attributed to the higher energy associated with UVB, suggesting that the protective efficacy of DNX varies between UVA and UVB radiations. Notably, a 49.21% elevation in viability was observed in the group pre-treated with 10 µM DNX relative to the control.

#### 3.2.3. Tyrosinase Activity

**Figure 4:**
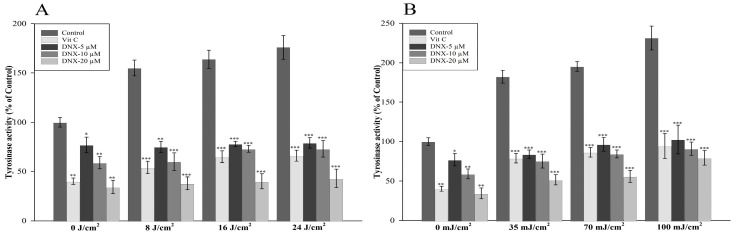
Activity of tyrosinase in HFF-1 cells exposed to UVA (**A**) and UVB (**B**) radiation was determined after treatment with varying concentrations of DNX. Statistical significance in this figure indicates the observed differences between the control group and the groups treated with varying concentrations of DNX and VitC, all subjected to the same UV dosage levels. The mean percentages ±  s.e.m. are shown (*n* = 6). * *p* < 0.05; ** *p* < 0.001; *** *p* < 0.005.

Upon exposure to 8 J/cm^2^ of UVA, tyrosinase activity surged to 155% in control HFF-1 cells. In contrast, a mere 38% increase was observed in cells fortified with 20 µM DNX. For the maximal UVA dose of 24 J/cm^2^, relative tyrosinase activity peaked at 176% in the control, while in the DNX-treated group, it rested at 43%. The most pronounced disparity relative to the control, at 133%, was noted for cells exposed to 20 µM DNX under 24 J/cm^2^ UVA (Figure 4A).

In the control group, tyrosinase activity registered its nadir at 34.2% with a 20 µM concentration of DNX, when juxtaposed against untreated cells or those devoid of VitC treatment. This observation suggests that both DNX and VitC can impede melanogenesis, even in the absence of UV exposure.

In UVB-exposed HFF-1 cells treated with DNX, the most subdued tyrosinase activity was detected in cells administered 20 µM DNX at an irradiation level of 35 mJ/cm^2^ UVB. The most considerable discrepancy when juxtaposed with the UVB-exposed control was a surge of 152% in the cohort treated with 20 µM DNX under 100 mJ/cm^2^ UVB. A cursory analysis of the mean tyrosinase activity indirectly hints at heightened melanogenesis in cells subjected to the more potent UVB dosage in comparison to UVA (Figure 4B).

#### 3.2.4. Superoxide Dismutase Activity

**Figure 5:**
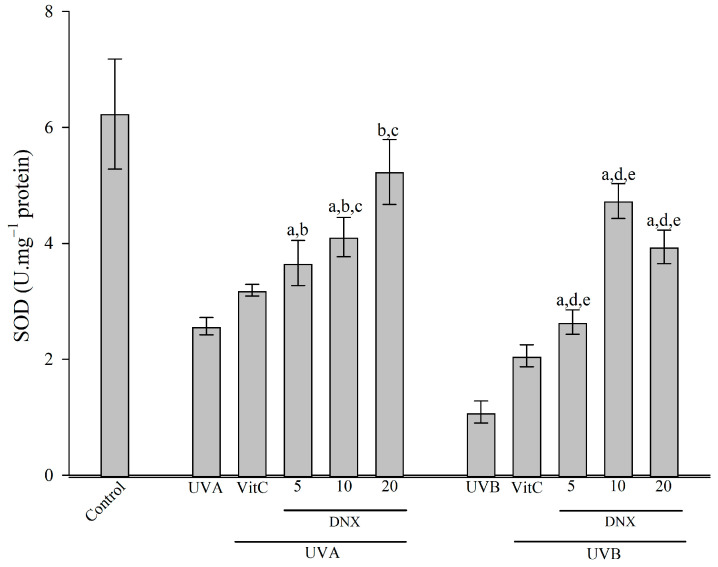
Activity of SOD in HFF-1 cell after UVA and UVB irradiation with different concentrations of DNX and VitC. Values are represented as mean ± standard error of mean. ^a^ *p* < 0.05 vs. control group. ^b^ *p* < 0.05 vs. UVA group. ^c^ *p* < 0.05 VitC-treated + UVA group. ^d^ *p* < 0.05 UVB group. ^e^ *p* < 0.05 VitC-treated + UVB group.

Referring to Figure 5, a marked reduction in SOD levels is evident in cells subjected solely to UVB (100 mJ/cm^2^) irradiation (1.09 ± 0.19 U/mg protein) when juxtaposed with the control group (6.23 ± 0.95 U/mg protein). This underscores the extent of cellular detriment. In cells treated with VitC, UVB irradiation resulted in a nearly two-fold augmentation in activity, registering at 2.06 ± 0.18 U/mg protein. Remarkably, the peak SOD activity was observed in the 10 µM DNX group, measuring 4.73 ± 0.3 U/mg protein, which approximates 75.9% of the values noted in the control group.

In cells subjected to UVA radiation, SOD activity experienced a significant decrease of 58.7%, amounting to 2.57 ± 0.15 U/mg protein when compared to the control. Conversely, when HFF-1 cells were treated with VitC, a rise of approximately 24.1% in SOD activity was recorded, resulting in a value of 3.19 ± 0.1 U/mg protein compared to those solely subjected to UVA exposure. Impressively, the zenith of SOD activity was identified in the 20 µM DNX group, measuring at 5.23 ± 0.56 U/mg protein, which is a substantial 103.5% increment when contrasted with the UVA-only treated group.

#### 3.2.5. ROS and MDA Content

**Figure 6:**
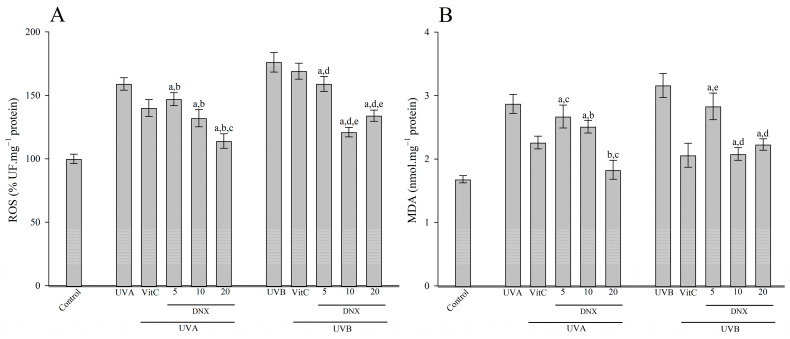
(**A**) ROS level in HFF-1 cells post UVA and UVB exposure, influenced by varying concentrations of DNX (**B**) MDA in HFF-1 cell after UVA and UVB irradiation with varying concentrations of DNX. Values are represented as mean ± standard error of mean. ^a^ *p* < 0.05 vs. control group. ^b^ *p* < 0.05 vs. UVA group. ^c^ *p* < 0.05 VitC-treated + UVA group. ^d^ *p* < 0.05 UVB group. ^e^ *p* < 0.05 VitC-treated + UVB group.

As depicted in Figure 6A, when HFF-1 cells were exposed to UVB radiation, they exhibited increased levels of ROS (176 ± 7.7%) and MDA (3.16 ± 0.19 nmol/mg protein) compared to cells not subjected to irradiation (ROS: 100 ± 3.6%; MDA: 1.68 ± 0.06 nmol/mg protein, respectively). In HFF-1 cells exposed to UVB radiation, treatment with VitC led to a significant decrease in ROS values (to 169 ± 6.3%) and MDA levels (to 2.06 ± 0.19 nmol/mg protein). Remarkably, the best inhibition effects of ROS elevation (134 ± 4.4%) and MDA (2.23 ± 0.09 nmol/mg protein) levels were observed in the 20 µM DNX group when contrasted with the UVB-only exposed cells.

Exposure of HFF-1 cells to UVA radiation increased both the ROS (159 ± 4.9%) and MDA (2.87 ± 0.15 nmol/mg protein) content in comparison to control cells (ROS: 100 ± 3.6%; MDA:1.68 ± 0.06 nmol/mg protein, respectively). On the other hand, HFF-1 cells treated with VitC resulted in a reduction in both ROS (140 ± 6.7%) and MDA (2.26 ± 0.10 nmol/mg protein) content compared to those only exposed to UVA. Interestingly, 20 µM DNX was found to inhibit the elevation in ROS (114 ± 5.7%) and MDA (1.83 ± 0.15 nmol/mg protein) concentration caused by UVA (Figure 6).

#### 3.2.6. Hydroxyproline Measurement

**Figure 7:**
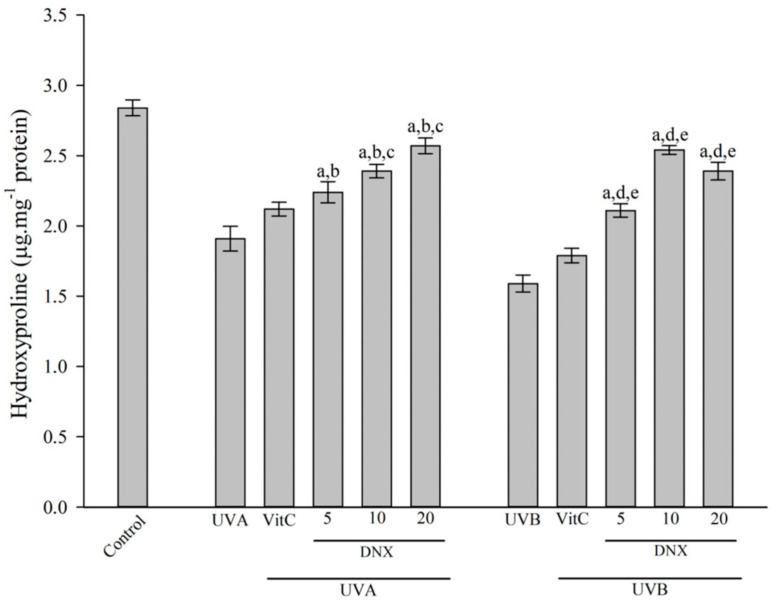
Hydroxyproline formation in HFF-1 cell after UVA and UVB irradiation with varying concentrations of DNX and VitC. Values are represented as mean ± standard error of mean. ^a^ *p* < 0.05 vs. control group. ^b^ *p* < 0.05 vs. UVA group. ^c^ *p* < 0.05 VitC-treated + UVA group. ^d^ *p* < 0.05 UVB group. ^e^ *p* < 0.05 VitC-treated + UVB group.

The amount of hydroxyproline present in the cells can be used to determine the extent of collagen loss. According to Figure 7, the HFF-1 cells exposed to UVB radiation (1.59 ± 0.061 µg/mg protein) were found to decrease hydroxyproline levels compared with those without irradiation (2.84 ± 0.057 µg/mg protein). The reduction in hydroxyproline content caused by UVB radiation in HFF-1 cells was inhibited by treatment with VitC (1.79 ± 0.052 µg/mg protein). Especially, it was determined that the application of 10 µM DNX (2.54 ± 0.031 µg/mg protein) in HFF-1 cells under UVB conditions found the highest increase in hydroxyproline compared with only UVB conditions (Figure 7).

When the cells were exposed to UVA radiation, the cells were observed to have reduced hydroxyproline levels (1.91 ± 0.088 µg/mg protein) compared with those without irradiation (2.84 ± 0.057 µg/mg protein). The UVA-irradiated HFF-1 cells underwent an increase in hydroxyproline with the addition of VitC (2.12 ± 0.049 µg/mg protein). However, the most significant elevation in hydroxyproline levels was observed in HFF-1 cells exposed to 20 µM DNX concentration under UVA radiation (2.57 ± 0.055 µg/mg protein) compared with the UVA-only treated cells.

#### 3.2.7. Wound Healing Assay

**Figure 8:**
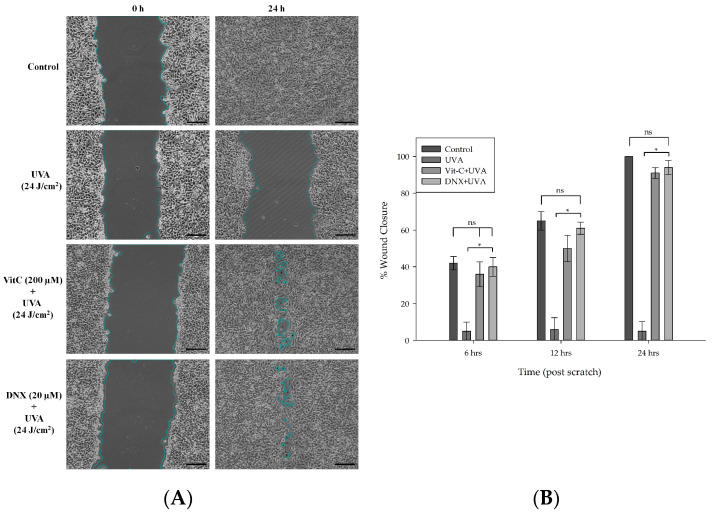
Impact of DNX on the wound healing response in UVA-exposed HFF-1 cells. (**A**) DNX counteracts the impediments in wound healing caused by UVA exposure. Upon reaching confluency, cells were scratched using a pipette tip and subsequently pretreated with DNX and VitC before UVA exposure. Inverted images (40× magnifications, 400 µm scale bar) of the scratched HFF-1 cells were captured at the 0 and 24 h marks post irradiation. (**B**) The wound healing assay’s graph, representing % wound closure, was generated by analyzing the captured inverted images through the Image-Pro Plus 6.0 Software. Data are expressed as the mean ± standard deviation of three independent experiments. Ns: non-significance, * *p* < 0.05.

**Figure 9:**
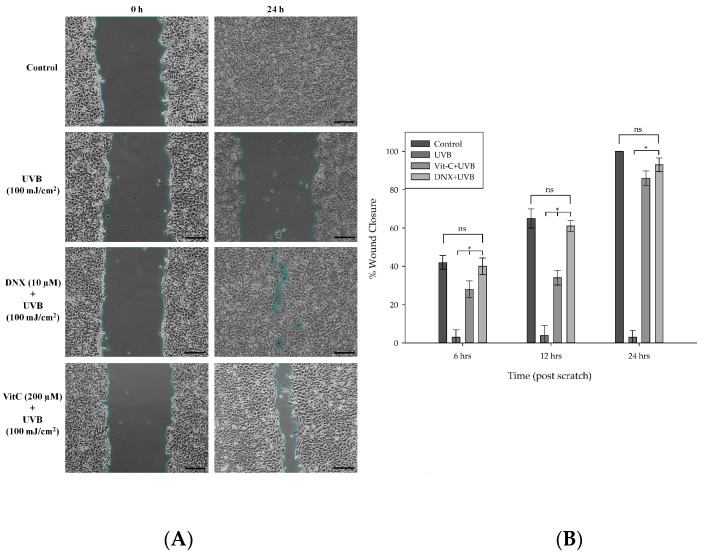
Effect of DNX on scratch wound healing in UVB-irradiated HFF-1 cells. (**A**) DNX rescues the UVB-induced wound-healing defect. Confluent cells were mechanically scratched with a pipette tip and pretreated with DNX and VitC prior to UVB irradiation. Invert micrographs (40× magnifications, 400 µm scale bar) of scratched HFF-1 cells were taken at 0 and 24 h following irradiation. (**B**) In the wound healing assay, the graph of % wound closure was derived from the assessment of inverted micrographs using Image-Pro Plus 6.0 Software. Data are expressed as the mean ± standard deviation of three independent experiments. ns: non-significance, * *p* < 0.05.

Given that enhanced fibroblast migration correlates with advanced re-epithelialization and wound sealing, this study investigated the potential positive impact of DNX on the wound recovery process. Based on the findings obtained from MTT and flow cytometry experiments, the wound-healing assay was conducted using optimal DNX concentrations: 20 μM for UVA (Figure 8A) and 10 μM for UVB (Figure 9A). These concentrations demonstrated the highest cell viability. The efficacy of DNX was further evaluated under the most intense doses of UVA and UVB. The migratory capacity of HFF-1 cells was assessed using a scratch wound-healing assay post treatment with 20 μM of DNX for UVA and 10 μM for UVB over intervals of 0, 6, 12, and 24 h. Notably, there was a marked reduction in the initial wound area after administering DNX at concentrations of 10 μM for UVA and 20 μM for UVB, and this was observed in a time-dependent manner.

In HFF-1 cells treated with 20 µM DNX and subsequently exposed to 24 J/cm^2^ UVA, the wound closure percentage was observed to be 40% at 6 h (Figure 8B). However, no significant difference was discerned when juxtaposed with both the control and VitC cohorts. By the 12th and 24th hours, the rates of closure in the control and DNX groups were nearly identical. Statistically significant differences were apparent at the 12th and 24th hours, implying that DNX may promote migration and proliferation more effectively than the VitC group. This was evidenced by closure percentages of 61% and 94% for the DNX group, compared to 50% and 91% for the VitC group, respectively. Furthermore, the lack of wound closure progression in the UVA-only group was anticipated.

It can be inferred that cells exposed to UVB exhibited relatively reduced wound closure compared to those exposed to UVA, likely because UVB possesses higher energy than UVA. In HFF-1 cells subjected to DNX and VitC treatments, a notable difference was observed at both the 6 and 12 h mark. Closure percentages of 40% and 61% were noted in the DNX-treated cells, and 28% and 34% in the VitC-treated cells, respectively. Furthermore, no significant disparities were detected between the control group (with closure rates of 42% and 65% for the respective hours) and the DNX group during these time intervals. At 12 h, no statistically significant differences were found among the control, VitC, and DNX groups (Figure 9B). Furthermore, in cells exposed solely to UVB, no difference in wound closure percentages was observed over 24 h, mirroring the findings in cells exposed only to UVA.

## 4. Discussion

Deinoxanthin, a derivative of xanthophyll, has emerged as a compound of significant interest in recent research, particularly in the realm of health and beauty [40,41,42,43,44,45]. As a member of the oxygenated carotenoids, xanthophylls are recognized for their unique structural features, such as terminal ketones, hydroxy groups, epoxy, and aldehyde groups. These characteristics afford them superior radical scavenging activity compared to carotenes like lycopene and β-carotene. Notably, DNX boasts a high antioxidant capacity, playing a crucial role in the resistance of *D. radiodurans*, a notable extremophile, to radiation [8].

The antioxidant capability of DNX, which exceeds that of lycopene, β-carotene, and zeaxanthin, becomes especially significant in comprehending the cellular resilience of D. radiodurans when exposed to oxidative stress. Moreover, recent findings hint at the potential role of *D. radiodurans*, particularly its cell wall component known as DeinoWall, in modulating immunity and possibly mitigating allergic inflammatory responses, although the exact mechanisms remain to be elucidated [11]. Given this backdrop, the focus of our ongoing investigation revolves around DNX, a key constituent of DeinoWall, and its protective effects on human skin cells exposed to ultraviolet radiation.

In the current investigation, the MTT assay was employed to gauge the protective potential of varying DNX concentrations on HFF-1 cells under UVB and UVA irradiation conditions. It was observed that HFF-1 cell viability experienced a significant decline under different UVB exposures (Figure 2B). Interestingly, cells exposed to UVB and subsequently treated with DNX exhibited variable resilience levels. For instance, at 35 mJ/cm^2^ UVB exposure, the treatment with 5 µM DNX yielded the highest viability relative to the control, while at 100 mJ/cm^2^, the most marked statistical difference in cell viability was seen in the 10 µM DNX treated cells. It was further deduced that in trials involving UVB radiation at an intensity of 100 mJ/cm^2^, DNX demonstrated superior protective capabilities than VitC. This inference was also supported by subsequent evaluations under UVA irradiation conditions, where DNX consistently outperformed VitC in maintaining cell viability.

In dermal layers, fibroblasts play a pivotal role in generating and distributing key extracellular matrix components such as collagen and elastin [46]. Among these, the Human Foreskin Fibroblast-1 (HFF-1) cells, a significant subset of fibroblasts, hold particular importance in human dermal investigations. Though numerous studies have centered on skin cancer and photoaging, literature addressing the specific effects of ultraviolet exposure on HFF-1 cells is relatively sparse, leaving the exact implications of UV exposure on their survival and physiologic behavior largely elusive.

UVB radiation predominantly targets the skin’s superficial layers, causing noteworthy damage. Studies have corroborated that it results in erythema and direct modifications to DNA, consequently augmenting the susceptibility to skin cancers, predominantly basal cell and squamous cell carcinomas [46,47,48,49,50]. In contrast, UVA radiation, while considered less damaging superficially than UVB, penetrates deeper into skin tissues, influencing collagen synthesis and structural integrity [50,51].

The antioxidant property of Astaxanthin has been widely acknowledged, with its efficacy surpassing other carotenoids such as lutein, canthaxanthin, and beta-carotene [52,53,54,55,56]. A notable study by Chung et al. (2018) explored UVB radiation’s detrimental effects on HaCaT keratinocytes, especially concerning skin aging, extracellular matrix degradation, and wrinkle formation [57]. Surprisingly, under similar UVB dosage conditions, DNX exhibited a remarkable average cell viability of 77% at only 5 µM concentration, as opposed to a 100 µM concentration of astaxanthin, which achieved around 50% viability.

The MTT assay used in this study elucidated how DNX provides protection to HFF-1 cells from UVA and UVB radiation. When exposed to UVB radiation, notably at 100 mJ/cm^2^, all DNX concentrations tested offered more cellular protection than VitC. Furthermore, in UVA exposure scenarios, cells treated with DNX consistently outperformed those treated with other compounds. Especially significant was the observation that HFF-1 cells pre-treated with DNX maintained higher viability rates under both UVA and UVB radiation compared to controls and other treatment groups.

In light of a study examining the photoprotective capabilities of Citrus sinensis extract on HFF-1 and NCTC 2544 cell lines, the extract’s performance was underwhelming, showing around 40% vitality at a UVA dosage of 15 J/cm^2^ [58]. In contrast, DNX showcased a more robust protective effect, presenting an average viability rate close to 45% under comparable UVA conditions. These results highlight DNX’s potential as an exceptional photoprotective agent and underscore the importance of conducting more research to explore its advantageous effects.

In the discussion of apoptotic effects induced by UV radiation on HFF-1 cells, certain findings from the current study resonate with the existing literature. Sunburn cells, representative keratinocytes undergoing apoptosis post UV exposure, are seen as a protective measure, eliminating potentially malignantly transformed cells [52]. Such a mechanism has been detailed in a plethora of studies, particularly emphasizing UV radiation’s apoptotic impacts, evaluated primarily through the Annexin V-FITC/PI method via flow cytometry [59,60,61,62,63,64].

In the current investigation, to evaluate the anti-apoptotic efficacy of DNX, the maximal selected doses of UVA and UVB (24 J/cm^2^ and 100 mJ/cm^2^, respectively) were utilized. The overarching aim was to gauge DNX’s utmost protective potential across the designated concentration range. A marked decrease in cell viability was observed under DNX, paralleling the results acquired from the MTT analysis. Interestingly, such effects can potentially be ascribed to DNX’s pro-apoptotic attributes, as delineated in the previous literature [40,41,45,65]. Despite this, in a control setting, HFF-1 cell viability remained high at 91% with a 20 µM DNX concentration. Furthermore, cells exposed to 24 J/cm^2^ UVA and pre-treated with 20 µM DNX showcased peak viability at 81.13%, denoting a substantial 33.42% increase in comparison to the control group. Such findings, mirroring the outcomes from the MTT assessment, reiterate the internal consistency of the study.

The utilization of the Annexin V-FITC/PI double-staining assay further detailed the protective prowess of DNX against apoptosis induced by UVA and UVB in HFF-1 cells. Following exposure to defined UVA and UVB intensities, the post irradiation apoptotic manifestations were quantified. In scenarios involving 100 mJ/cm^2^ UVB exposure, HFF-1 cells pre-treated with 10 µM DNX revealed an optimal viability of 74.55%. In its absence, a stark decrease to 25.34% was noted, with the early and late apoptotic phases accounting for 3.59% and 69.12%, respectively. An intriguing aspect was the diminished cell viability at 20 µM DNX under UVB compared to UVA exposure, insinuating a difference in DNX’s protective capability against UVA and UVB, possibly due to UVB’s heightened energy. It is worth mentioning that there was a significant increase in viability, specifically a 49.21% improvement, observed in the group pre-treated with 10 µM DNX when compared to the control group.

The intricate process of melanin synthesis in skin involves both enzymatic and non-enzymatic pathways [66,67]. Tyrosinase, a copper-rich monooxygenase enzyme, stands as the initial facilitator in this cascade, converting L-tyrosine into L-3,4-dihydroxyphenylalanine (L-DOPA). The subsequent transformation of L-DOPA into L-dopaquinone is also orchestrated by tyrosinase, with the final result being melanin formation [68]. An amplified rate of melanogenic reactions is observed when substrate levels, such as L-tyrosine and L-DOPA, elevate. Moreover, melanocyte proliferation is often accelerated by UV exposure, consequently intensifying melanin synthesis.

Considering the essential role of UV exposure in stimulating melanogenesis, it becomes vital to conduct assessments focused on inhibiting melanogenesis. This is necessary for identifying substances that can reduce the negative effects of UV-induced skin damage. The primary target is often to suppress tyrosinase activity and the associated substrates, namely L-tyrosine and L-DOPA. Xanthophylls, a subset of oxygenated carotenoids, have garnered attention due to their health and beauty associations. Their distinctive structural features offer enhanced antioxidant properties compared to carotenes such as lycopene and β-carotene [69,70,71]. DNX, known for its robust antioxidant properties, is acknowledged for being a potent ROS scavenger, even surpassing lycopene, β-carotene, astaxanthin, and zeaxanthin in this aspect. This antioxidant property of DNX contributes significantly to the resilience of *D. radiodurans* under oxidative stress [40,41,42,43,44,45]. It was discovered in a study conducted on cultured normal human melanocytes that pre-treatment with Astaxanthin interrupted tyrosinase activity when stimulated by the stem cell factor (SCF) but had no effect when stimulated by endothelin-1. Additionally, no direct inhibitory effect on in vitro tyrosinase activity by Astaxanthin was observed. From these findings, it can be inferred that pigmentation stimulated by SCF is attenuated by Astaxanthin through the direct interruption of SCF-associated intracellular signaling linkages. This leads to an increased expression of MITF, culminating in the stimulated expression of melanogenic genes and proteins through a mechanism that is independent of reactive oxygen species depletion [72].

In this study, upon exposure to 8 J/cm^2^ of UVA, a pronounced increase in tyrosinase activity to 155% was recorded in control HFF-1 cells. However, this surge was restrained to 38% in cells fortified with 20 µM DNX. Similarly, when exposed to the maximal UVA dose of 24 J/cm^2^, the relative tyrosinase activity was found to escalate to 176% in the control, whereas it was limited to 43% in the DNX-treated group (Figure 4A). Intriguingly, a reduction in tyrosinase activity to 34.2% was observed in the control group when administered with a 20 µM DNX concentration. Such findings allude to the potential of DNX, and even VitC, in thwarting melanogenesis, irrespective of UV exposure. Previous research has endorsed the melanogenesis-inhibiting prowess of VitC, proposing mechanisms such as its ROS-quenching potential, its influence on α-melanocyte-stimulating hormones (α-MSH), or its ability to acidify intracellular melanocytes [73,74,75,76].

SOD, renowned for its potent free radical neutralizing capacity, has been established as a fundamental component in combatting photo-oxidative stress. The quantitative assessment of SOD levels has been considered an instrumental marker to determine the magnitude of oxidative cellular damage [75]. Within mammalian cells, two prominent SOD isoforms, manganese-SOD (Mn-SOD) and copper,zinc-SOD (Cu,Zn-SOD), have been identified and both have been detected in keratinocytes [77,78]. These isozymes are attributed with distinct physiological functions and have been associated with different disorders within biological systems.

In the present study, an analysis of Figure 5 revealed that cells exposed solely to UVB (100 mJ/cm^2^) irradiation experienced a significant reduction in SOD levels, registering at 1.09 ± 0.19 U/mg protein, in contrast to the control group’s 6.23 ± 0.95 U/mg protein. This decline accentuates the considerable cellular oxidative damage. Intriguingly, in cells subjected to UVB and treated with VitC, SOD activity was observed to have nearly doubled, reaching 2.06 ± 0.18 U/mg protein. The zenith of SOD activity, however, was detected in the group treated with 10 µM DNX, amounting to 4.73 ± 0.3 U/mg protein, which approached 75.9% of the control group values. Similarly, UVA-exposed cells demonstrated a decrease in SOD activity by 58.7%, equating to 2.57 ± 0.15 U/mg protein. Yet, when these UVA-exposed HFF-1 cells were supplemented with VitC, a surge in SOD activity by approximately 24.1% was recorded, culminating in a value of 3.19 ± 0.1 U/mg protein. Notably, the pinnacle of SOD activity in the context of UVA exposure was noted in the 20 µM DNX group, reaching 5.23 ± 0.56 U/mg protein.

Previous studies have documented changes in the activity of SOD isozymes following exposure to UVB radiation. Specificly, human keratinocyte cultures demonstrated an immediate increase in Cu,Zn-SOD activity following a singular UVB exposure. In contrast, the Mn-SOD activity was noted to decrease after UVB exposure before eventually reverting to baseline [79]. This suggests the possibility of contrasting roles of these SOD isozymes in defending against UVB-induced oxidative stress. Moreover, studies examining Astaxanthin’s protective efficacy against UVA have spotlighted a notable upsurge in SOD activity in the group exposed to UVA compared to the controls [80,81].

UV radiation’s capability to instigate the excessive production of ROS, subsequently undermining skin barrier function, has been well documented [82]. The harm inflicted by ROS on the membrane precipitates the generation of primary and secondary lipid peroxidation products [83]. Importantly, MDA, an end-product of lipid peroxidation, has been firmly linked with oxidative skin damage elicited by UV radiation [82,83]. To quantify the oxidative assault imposed by UVB and UVA in HFF-1 cells, the present study measured total ROS and MDA content.

In this context, Figure 6A delineated that upon UVB radiation exposure, ROS and MDA levels in HFF-1 cells were observed to be elevated (176 ± 7.7% and 3.16 ± 0.19 nmol/mg protein, respectively) when juxtaposed with non-irradiated cells (ROS: 100 ± 3.6%; MDA: 1.68 ± 0.06 nmol/mg protein). Notably, these escalated values of ROS (169 ± 6.3%) and MDA (2.06 ± 0.19 nmol/mg protein) were found to be significantly diminished in the presence of VitC under UVB conditions. Strikingly, the 20 µM DNX group displayed the most pronounced inhibitory effects on elevated ROS (2.23 ± 0.09%) and MDA (134 ± 4.4 nmol/mg protein) levels when contrasted with UVB-only exposed cells. In contrast, UVA radiation’s exposure resulted in an uptick in both ROS (159 ± 4.9%) and MDA (2.87 ± 0.15 nmol/mg protein) vis-a-vis the control cells (ROS: 100 ± 3.6%; MDA:1.68 ± 0.06 nmol/mg protein). Conversely, the presence of VitC instigated a decrement in ROS (140 ± 6.7%) and MDA (2.26 ± 0.10 nmol/mg protein) content relative to cells merely exposed to UVA. Intriguingly, the 20 µM DNX group demonstrated a potent inhibitory effect on ROS (114 ± 5.7%) and MDA (1.83 ± 0.15 nmol/mg protein) concentrations triggered by UVA (as presented in Figure 6B).

Highlighting the significance of xanthophylls, a sub-category of oxygenated carotenoids, their unique structural attributes render them superior radical scavengers in comparison to other carotenes such as β-carotene and lycopene [69,70,71,72]. DNX, particularly recognized for its powerful antioxidant qualities, surpasses even lycopene, β-carotene, and zeaxanthin as a formidable ROS scavenger. Such antioxidative prowess significantly bolsters *D. radiodurans*’ resilience against oxidative duress [40,41,42,43,44,45].

The detrimental impacts of persistent UV radiation exposure on the skin are well-recognized, predominantly accelerating aging by enhancing collagen degradation and prompting epidermal thickening [84]. This photodamage becomes apparent not only through changes in the skin’s texture but also in its decreased elasticity, ultimately contributing to the development of wrinkles. Central to this phenomenon is the decline in collagen type I concentrations, a fundamental component of the skin’s dermal layer. Consistent with these findings, the present study has evaluated the hydroxyproline content in cells as an indicative marker for collagen loss following prolonged UVB exposure [85]. In the HFF-1 cells, when exposed to UVB radiation, a notable decrease in hydroxyproline levels (1.59 ± 0.061 µg/mg protein) was observed in comparison to non-irradiated counterparts (2.84 ± 0.057 µg/mg protein). Interestingly, this depletion was mitigated by VitC treatment (1.79 ± 0.052 µg/mg protein), with 10 µM DNX application under UVB conditions showing the most significant enhancement in hydroxyproline content (2.54 ± 0.031 µg/mg protein). A similar trend was evident upon UVA exposure, with notable reductions in hydroxyproline (1.91 ± 0.088 µg/mg protein), yet a marked elevation was observed with 20 µM DNX application under UVA conditions (2.57 ± 0.055 µg/mg protein). The pivotal role of hydroxyproline, a core collagen constituent in the skin, has been documented in seminal studies [86,87]. The major synthetic amino acid in collagen tissues, hydroxyproline, further accentuates its value, offering a nuanced understanding of collagen content shifts. The prevalence of Procollagen 1 further highlights its position as the predominant fibrous collagen variant in human physiology. In a recent investigation, the therapeutic potential of ζ-carotene-like compounds (CLC) was examined, particularly focusing on its ability to mitigate the deleterious effects of UVB radiation on the skin. Notably, when administered topically, CLC not only showcased profound antioxidant properties but also significantly enhanced the hydroxyproline and Procollagen 1 content, suggesting a protective role against collagen degradation. Through a myriad of techniques, ranging from qRT-PCR to immunohistochemistry staining, the study further underscored CLC’s potential in regulating inflammatory markers, bolstering cell viability, and attenuating melanin synthesis. This aligns with the notion that certain carotenoid derivatives may hold promise in skin photoprotection and repair [88].

In the intricate progression of typical skin wound recovery, one can identify specific stages, including hemostasis, inflammation/granulation, re-epithelialization, and contraction/tissue restructuring [89,90,91]. The integration of various biological and molecular events, including cellular proliferation, migration, and extracellular matrix production, is essential for the seamless progression through these phases. An imbalance in these events might lead to delayed healing or even result in chronic, nonhealing wounds [92,93].

Keratinocyte migration, closely related to the formation and disassembly of cellular adhesion sites and cytoskeletal restructuring, is fundamental to wound healing. Regulated by the Rho family of signaling molecules, like Cdc42, Rac1, and RhoA, this process has garnered significant attention in recent studies. For instance, the role of astaxanthin, derived from L. vannamei, in enhancing keratinocyte migration has been demonstrated, echoing previous findings in cultured HBMEC and RASMC. The broader roles of the Rho family, beyond just migration, include cell cycle progression and differentiation [94,95,96,97]. With the astaxanthin’s potential influence on these processes, there are optimistic implications for wound healing. Similarly, a relationship between increased fibroblast migration and re-epithelialization has been established. Within this context, the impact of DNX on the wound healing process was evaluated. Results from MTT and flow cytometry experiments dictated the optimal DNX concentrations for subsequent wound-healing assays (20 μM for UVA and 10 μM for UVB), and these concentrations were found to ensure maximum cell viability. Assessments were conducted on HFF-1 cell migration post-DNX treatment, revealing a time-dependent reduction in the original wound area for specific concentrations. Notably, in HFF-1 cells treated with 20 μM DNX, a wound closure percentage of 40% was observed at 6 h post UVA exposure. However, the differences were not as pronounced when compared to the control and VitC groups, especially during longer-term assessments. Valuable insights were gleaned from the cells exposed to UVB, where diminished wound closure was noted, likely due to the higher energy of UVB radiation compared to UVA. Ultimately, these findings underscored the potential of DNX in cell migration and its therapeutic potential in wound healing, drawing a parallel to the impact of compounds like astaxanthin in similar situations.

In this study, preliminary insights were gained into the protective effects of DNX on skin cells through in vitro findings. While these results are significant, it is important to acknowledge the inherent limitations of such models. In vitro studies, despite their strengths, cannot fully replicate the intricate interactions and physiological contexts present in living organisms.

Given this, the cellular benefits of DNX observed in this study might not directly translate to in vivo conditions on human skin. It is anticipated that future investigations, possibly involving animal models or human clinical trials, would further validate and expand upon these findings. These studies could provide a deeper understanding of the efficacy and safety of DNX in realistic settings.

This research emphasizes the protective effects of DNX, laying a foundation for exploring the broader potential of carotenoids in skincare. However, with a myriad of naturally occurring carotenoids, including well-documented ones like Beta-carotene, Lutein, Zeaxanthin, Lycopene, Astaxanthin, Canthaxanthin, and Fucoxanthin, there is a clear impetus for further research. Future studies that incorporate comparisons with these renowned carotenoids would provide a comprehensive perspective on their skincare capabilities.

Furthermore, while the cellular advantages of DNX have been underscored, challenges associated with its practical application in skincare or therapeutic contexts cannot be overlooked. A significant concern pertains to the stability of DNX, especially its vulnerability to environmental factors like light and temperature. Additionally, the bioavailability of DNX, whether applied topically or ingested, warrants meticulous evaluation. The efficacy of an active ingredient largely hinges on its absorption and utilization by the skin or body.

It is equally crucial to understand DNX’s compatibility with other ingredients in skincare formulations. Interactions with other compounds could significantly affect the overall safety and efficacy of the resultant product. Like all active ingredients, potential side effects of DNX, be it short-term or long-term, require thorough investigation to safeguard end users. In summation, while the initial insights on DNX are optimistic, an exhaustive evaluation concerning its stability, bioavailability, formulation compatibility, and possible adverse effects is paramount prior to its widespread incorporation in skincare or therapeutic products.

## 5. Conclusions

In conclusion, our research suggests that DNX may offer potential as a photoprotective agent against UV-induced cellular damage. Preliminary findings indicate that it has the potential to support cell viability, moderate melanogenesis, increase SOD activity, and potentially mitigate oxidative stress. These results imply that DNX’s effects warrant further study in the fields of dermatology and cosmetology.

## Data Availability

Not applicable.

## Supplementary Materials

The following supporting information can be downloaded at: https://www.mdpi.com/article/10.3390/cimb45100528/s1.

## References

[1] Collaboration P., Ade P.A.R., Aghanim N., Alves M.I.R., Armitage-Caplan C., Arnaud M., Ashdown M., Atrio-Barandela F., Aumont J., Aussel H. (2014). Planck 2013 results. I. Overview of products and scientific results. Astron. Astrophys..

[2] Kochhar N., I․K K., Shrivastava S., Ghosh A., Rawat V.S., Sodhi K.K., Kumar M. (2022). Perspectives on the microorganism of extreme environments and their applications. Curr. Res. Microb. Sci..

[3] Koklesova L., Liskova A., Samec M., Buhrmann C., Samuel S.M., Varghese E., Ashrafizadeh M., Najafi M., Shakibaei M., Büsselberg D. (2020). Carotenoids in Cancer Apoptosis—The Road from Bench to Bedside and Back. Cancers.

[4] Darvin M.E., Lademann J., von Hagen J., Lohan S.B., Kolmar H., Meinke M.C., Jung S. (2022). Carotenoids in Human Skin In Vivo: Antioxidant and Photo-Protectant Role against External and Internal Stressors. Antioxidants.

[5] McArdle F., Rhodes L.E., Parslew R.A., Close G.L., Jack C.I., Friedmann P.S., Jackson M.J. (2004). Effects of oral vitamin E and β-carotene supplementation on ultraviolet radiation–induced oxidative stress in human skin. Am. J. Clin. Nutr..

[6] Reis-Mansur M.C.P., Cardoso-Rurr J.S., Silva J.V.M.A., de Souza G.R., Cardoso V.d.S., Mansoldo F.R.P., Pinheiro Y., Schultz J., Lopez Balottin L.B., da Silva A.J.R. (2019). Carotenoids from UV-resistant Antarctic *Microbacterium* sp. LEMMJ01. Sci. Rep..

[7] Milani A., Basirnejad M., Shahbazi S., Bolhassani A. (2017). Carotenoids: Biochemistry, pharmacology and treatment. Br. J. Pharmacol..

[8] Lim S., Jung J.-H., Blanchard L., de Groot A. (2019). Conservation and diversity of radiation and oxidative stress resistance mechanisms in *Deinococcus* species. FEMS Microbiol. Rev..

[9] Farci D., Slavov C., Tramontano E., Piano D. (2016). The S-layer protein DR_2577 binds deinoxanthin and under desiccation conditions protects against UV-radiation in *Deinococcus radiodurans*. Front. Microbiol..

[10] Reichrath J., Gordon-Thomson C., Tongkao-on W., Song E.J., Carter S.E., Dixon K.M., Mason R.S. (2014). Protection from ultraviolet damage and photocarcinogenesis by vitamin D compounds. Sunlight Vitam. D Ski. Cancer.

[11] Chen F., Ji H.J., Choi J.-I., Han S.H., Lim S., Seo H.S., Ahn K.B. (2022). Anti-allergic function of the cell wall (DeinoWall) from *Deinococcus radiodurans*. Mol. Immunol..

[12] Moens E., Veldhoen M. (2011). Epithelial Barrier Biology: Good Fences Make Good Neighbours. Immunology.

[13] Liu L., Song G., Song Z. (2022). Intrinsic Atopic Dermatitis and Extrinsic Atopic Dermatitis: Similarities and Differences. Clin. Cosmet. Investig. Dermatol..

[14] Feng S., Song G., Liu L., Liu W., Liang G., Song Z. (2022). Allergen-specific Immunotherapy Induces monocyte-derived Dendritic Cells but Attenuates Their Maturation and Cytokine Production in the Lesional Skin of an Atopic Dermatitis Mouse Model. J. Dermatol..

[15] Mullenders L.H. (2018). Solar UV damage to cellular DNA: From mechanisms to biological effects. Photochem. Photobiol. Sci..

[16] Salminen A., Kaarniranta K., Kauppinen A. (2022). Photoaging: UV radiation-induced inflammation and immunosuppression accelerate the aging process in the skin. Inflamm. Res..

[17] Marabini L., Melzi G., Lolli F., Dell’Agli M., Piazza S., Sangiovanni E., Marinovich M. (2020). Effects of *Vitis vinifera* L. leaves extract on UV radiation damage in human keratinocytes (HaCaT). J. Photochem. Photobiol. B Biol..

[18] Nichols J.A., Katiyar S.K. (2010). Skin photoprotection by natural polyphenols: Anti-inflammatory, antioxidant and DNA repair mechanisms. Arch. Dermatol. Res..

[19] Anbualakan K., Tajul Urus N.Q., Makpol S., Jamil A., Mohd Ramli E.S., Md Pauzi S.H., Muhammad N. (2022). A Scoping Review on the Effects of Carotenoids and Flavonoids on Skin Damage Due to Ultraviolet Radiation. Nutrients.

[20] Gromkowska-Kępka K.J., Puścion-Jakubik A., Markiewicz-Żukowska R., Socha K. (2021). The impact of ultraviolet radiation on skin photoaging—Review of in vitro studies. J. Cosmet. Dermatol..

[21] Lu W., Kong C., Cheng S., Xu X., Zhang J. (2023). Succinoglycan riclin relieves UVB-induced skin injury with anti-oxidant and anti-inflammatory properties. Int. J. Biol. Macromol..

[22] Kong S.-Z., Li D.-D., Luo H., Li W.-J., Huang Y.-M., Li J.-C., Hu Z., Huang N., Guo M.-H., Chen Y. (2018). Anti-photoaging effects of chitosan oligosaccharide in ultraviolet-irradiated hairless mouse skin. Exp. Gerontol..

[23] Pillaiyar T., Manickam M., Jung S.-H. (2017). Recent development of signaling pathways inhibitors of melanogenesis. Cell. Signal..

[24] Guan L.L., Lim H.W., Mohammad T.F. (2021). Sunscreens and photoaging: A review of current literature. Am. J. Clin. Dermatol..

[25] He H., Li A., Li S., Tang J., Li L., Xiong L. (2020). Natural components in sunscreens: Topical formulations with sun protection factor (SPF). Biomed. Pharmacother..

[26] Narla S., Lim H.W. (2020). Sunscreen: FDA regulation, and environmental and health impact. Photochem. Photobiol. Sci..

[27] Schneider S.L., Lim H.W. (2019). Review of environmental effects of oxybenzone and other sunscreen active ingredients. J. Am. Acad. Dermatol..

[28] Badmus U.O., Ač A., Klem K., Urban O., Jansen M.A. (2022). A meta-analysis of the effects of UV radiation on the plant carotenoid pool. Plant Physiol. Biochem..

[29] Kılınç S. (2019). Purification and Optimization of Deinoxanthin Isolated from Deinococcus Radiodurans R1. Master’s Thesis.

[30] Saqib S., Nazeer A., Ali M., Zaman W., Younas M., Shahzad A., Sunera, Nisar M. (2022). Catalytic Potential of Endophytes Facilitates Synthesis of Biometallic Zinc Oxide Nanoparticles for Agricultural Application. BioMetals.

[31] Kawashima S., Funakoshi T., Sato Y., Saito N., Ohsawa H., Kurita K., Nagata K., Yoshida M., Ishigami A. (2018). Protective effect of pre-and post-vitamin C treatments on UVB-irradiation-induced skin damage. Sci. Rep..

[32] Savini I., D’Angelo I., Ranalli M., Melino G., Avigliano L. (1999). Ascorbic acid maintenance in HaCaT revents radical formation and apoptosis by UV-B. Free Radic. Biol. Med..

[33] Noh D., Choi J.G., Huh E., Oh M.S. (2018). Tectorigenin, a flavonoid-based compound of leopard lily rhizome, attenuates UV-B-induced apoptosis and collagen degradation by inhibiting oxidative stress in human keratinocytes. Nutrients.

[34] Kang J.S., Kim H.N., Kim J.E., Mun G.H., Kim Y.S., Cho D., Shin D.H., Hwang Y.-I., Lee W.J. (2007). Regulation of UVB-induced IL-8 and MCP-1 production in skin keratinocytes by increasing vitamin C uptake via the redistribution of SVCT-1 from the cytosol to the membrane. J. Investig. Dermatol..

[35] Wang J., Bian Y., Cheng Y., Sun R., Li G. (2020). Effect of lemon peel flavonoids on UVB-induced skin damage in mice. RSC Adv..

[36] Salgado-Pabón W., Schlievert P.M. (2016). Aortic valve damage for the study of left-sided, native valve infective endocarditis in rabbits. Superantigens Methods Protoc..

[37] McCord J.M., Fridovich I. (1969). Superoxide dismutase: An enzymic function for erythrocuprein (hemocuprein). J. Biol. Chem..

[38] Uchiyama M., Mihara M. (1978). Determination of malonaldehyde precursor in tissues by thiobarbituric acid test. Anal. Biochem..

[39] Zhang C., Lv J., Qin X., Peng Z., Lin H. (2022). Novel Antioxidant Peptides from Crassostrea Hongkongensis Improve Photo-Oxidation in UV-Induced HaCaT Cells. Mar. Drugs.

[40] Günay N., Kuzucu M. (2023). Agonistic Effects of Deinoxanthin on Tamoxifen Antiproliferative Activity on HER2 Positive Breast Cancer: An In Vitro Study on MDA-MB-453. Erzincan Univ. J. Sci. Technol..

[41] Tian B., Li J., Pang R., Dai S., Li T., Weng Y., Jin Y., Hua Y. (2018). Gold nanoparticles biosynthesized and functionalized using a hydroxylated tetraterpenoid trigger gene expression changes and apoptosis in cancer cells. ACS Appl. Mater. Interfaces.

[42] Farci D., Haniewicz P., de Sanctis D., Iesu L., Kereïche S., Winterhalter M., Piano D. (2022). The cryo-EM structure of the S-layer deinoxanthin-binding complex of Deinococcus radiodurans informs properties of its environmental interactions. J. Biol. Chem..

[43] Jeong S.-W., Kim J.-H., Kim J.-W., Kim C.Y., Kim S.Y., Choi Y.J. (2020). Metabolic engineering of extremophilic bacterium *Deinococcus radiodurans* for the production of the novel carotenoid deinoxanthin. Microorganisms.

[44] Asaka R., Ohshima K., Kawasaki S., Maoka T., Tode C., Wang-Otomo Z.-Y., Takaichi S. (2022). Major Carotenoids of *Meiothermus ruber* Are Deinoxanthin Glucoside Esters, Not Meiothermoxanthin Glucoside Esters. J. Nat. Prod..

[45] Yu S., Kim S., Kim J., Kim J.-W., Kim S.Y., Yeom B., Kim H., Choi W.I., Sung D. (2023). Highly Water-Dispersed and Stable Deinoxanthin Nanocapsule for Effective Antioxidant and Anti-Inflammatory Activity. Int. J. Nanomed..

[46] Han J.M., Song H.-Y., Jung J.-H., Lim S., Seo H.S., Kim W.S., Lim S.-T., Byun E.-B. (2023). Deinococcus radiodurans-derived membrane vesicles protect HaCaT cells against H_2_O_2_-induced oxidative stress via modulation of MAPK and Nrf2/ARE pathways. Biol. Proced. Online.

[47] Struwe M., Greulich K.-O., Junker U., Jean C., Zimmer D., Sutera W., Plappert-Helbig U. (2008). Detection of photogenotoxicity in skin and eye in rat with the photo comet assay. Photochem. Photobiol. Sci..

[48] Sheng J., Ding S., Liao H., Yao Y., Zhai Y., Zhan J., Wang X. (2023). Polyacrylonitrile/UV329/titanium oxide composite nanofibrous membranes with enhanced UV protection and filtration performance. RSC Adv..

[49] Agar N.S., Halliday G.M., Barnetson R.S., Ananthaswamy H.N., Wheeler M., Jones A.M. (2004). The basal layer in human squamous tumors harbors more UVA than UVB fingerprint mutations: A role for UVA in human skin carcinogenesis. Proc. Natl. Acad. Sci. USA.

[50] Barrette K., Zutterman N., Van Kelst S., Proby C., Garmyn M. (2013). Pattern of sensitivity of progressive cutaneous squamous cell carcinoma cells to UVB and oxidative stress-induced cell death. Photochem. Photobiol. Sci..

[51] Dazard J.-E., Gal H., Amariglio N., Rechavi G., Domany E., Givol D. (2003). Genome-wide comparison of human keratinocyte and squamous cell carcinoma responses to UVB irradiation: Implications for skin and epithelial cancer. Oncogene.

[52] Kidd P. (2011). Astaxanthin, cell membrane nutrient with diverse clinical benefits and anti-aging potential. Altern Med. Rev..

[53] O’Connor I., O’Brien N. (1998). Modulation of UVA light-induced oxidative stress by β-carotene, lutein and astaxanthin in cultured fibroblasts. J. Dermatol. Sci..

[54] Santocono M., Zurria M., Berrettini M., Fedeli D., Falcioni G. (2006). Influence of astaxanthin, zeaxanthin and lutein on DNA damage and repair in UVA-irradiated cells. J. Photochem. Photobiol. B Biol..

[55] Radrezza S., Carini M., Baron G., Aldini G., Negre-Salvayre A., D’Amato A. (2021). Study of Carnosine’s effect on nude mice skin to prevent UV-A damage. Free Radic. Biol. Med..

[56] Giampieri F., Alvarez-Suarez J.M., Tulipani S., Gonzàles-Paramàs A.M., Santos-Buelga C., Bompadre S., Quiles J.L., Mezzetti B., Battino M. (2012). Photoprotective potential of strawberry (Fragaria × ananassa) extract against UV-A irradiation damage on human fibroblasts. J. Agric. Food Chem..

[57] Chung Y.H., Jeong S.A., Choi H.S., Ro S., Lee J.S., Park J.K. (2018). Protective effects of ginsenoside Rg2 and astaxanthin mixture against UVB-induced DNA damage. Anim. Cells Syst..

[58] Tomasello B., Malfa G.A., Acquaviva R., La Mantia A., Di Giacomo C. (2022). Phytocomplex of a Standardized Extract from Red Orange (*Citrus sinensis* L. Osbeck) against Photoaging. Cells.

[59] Kulms D., Schwarz T. (2000). Molecular mechanisms of UV-induced apoptosis. Photodermatol. Photoimmunol. Photomed. Rev. Artic..

[60] Lee S.J., Kim J.E., Choi Y.J., Gong J.E., Park S.H., Douangdeuane B., Souliya O., Park J.M., Lee H.S., Kim B.-H. (2021). Therapeutic effects of Dipterocarpus tuberculatus with high antioxidative activity against UV-induced photoaging of NHDF cells and nude mice. Antioxidants.

[61] Lee S.J., Kim J.E., Choi Y.J., Jin Y.J., Roh Y.J., Seol A.Y., Song H.J., Park S.H., Uddin M.S., Lee S.W. (2022). Antioxidative role of *Hygrophila erecta* (Brum. F.) Hochr. on UV-induced photoaging of dermal fibroblasts and melanoma cells. Antioxidants.

[62] Guo W., An Y., Jiang L., Geng C., Zhong L. (2010). The protective effects of hydroxytyrosol against UVB-induced DNA damage in HaCaT cells. Phytother. Res. Int. J. Devoted Pharmacol. Toxicol. Eval. Nat. Prod. Deriv..

[63] Yang M.H., Hwang S.T., Um J.-Y., Ahn K.S. (2023). Cycloastragenol exerts protective effects against UVB irradiation in human dermal fibroblasts and HaCaT keratinocytes. J. Dermatol. Sci..

[64] Qu L., Wang F., Chen Y. (2023). Protective effect and mechanism research of *Phyllanthus emblica* Linn. fruit extract on UV-induced photodamage in keratinocytes. Photochem. Photobiol. Sci..

[65] Choi Y.-J., Hur J.-M., Lim S., Jo M., Kim D.H., Choi J.-I. (2014). Induction of apoptosis by deinoxanthin in human cancer cells. Anticancer Res..

[66] Pillaiyar T., Manickam M., Namasivayam V. (2017). Skin whitening agents: Medicinal chemistry perspective of tyrosinase inhibitors. J. Enzym. Inhib. Med. Chem..

[67] Park J., Jung H., Kim K., Lim K.M., Kim J.y., Jho E.h., Oh E.S. (2018). D-tyrosine negatively regulates melanin synthesis by competitively inhibiting tyrosinase activity. Pigment. Cell Melanoma Res..

[68] Slominski A., Zmijewski M.A., Pawelek J. (2012). L-tyrosine and L-dihydroxyphenylalanine as hormone-like regulators of melanocyte functions. Pigment. Cell Melanoma Res..

[69] Young A.J., Lowe G.L. (2018). Carotenoids—Antioxidant properties. Antioxidants.

[70] Mordi R.C., Ademosun O.T., Ajanaku C.O., Olanrewaju I.O., Walton J.C. (2020). Free radical mediated oxidative degradation of carotenes and xanthophylls. Molecules.

[71] Zaripheh S., Erdman J.W. (2002). Factors that influence the bioavailablity of xanthophylls. J. Nutr..

[72] Nakajima H., Fukazawa K., Wakabayashi Y., Wakamatsu K., Senda K., Imokawa G. (2012). Abrogating effect of a xanthophyll carotenoid astaxanthin on the stem cell factor-induced stimulation of human epidermal pigmentation. Arch. Dermatol. Res..

[73] Kim H.M., An H.S., Bae J.-S., Kim J.Y., Choi C.H., Kim J.Y., Lim J.H., Choi J.-h., Song H., Moon S.H. (2017). Effects of palmitoyl-KVK-L-ascorbic acid on skin wrinkles and pigmentation. Arch. Dermatol. Res..

[74] Miao F., Su M.-Y., Jiang S., Luo L.-F., Shi Y., Lei T.-C. (2019). Intramelanocytic acidification plays a role in the antimelanogenic and antioxidative properties of vitamin C and its derivatives. Oxidative Med. Cell. Longev..

[75] Shimada Y., Tai H., Tanaka A., Ikezawa-Suzuki I., Takagi K., Yoshida Y., Yoshie H. (2009). Effects of ascorbic acid on gingival melanin pigmentation in vitro and in vivo. J. Periodontol..

[76] Panich U., Tangsupa-A-Nan V., Onkoksoong T., Kongtaphan K., Kasetsinsombat K., Akarasereenont P., Wongkajornsilp A. (2011). Inhibition of UVA-mediated melanogenesis by ascorbic acid through modulation of antioxidant defense and nitric oxide system. Arch. Pharmacal Res..

[77] Deng H., Wan M., Li H., Chen Q., Li R., Liang B., Zhu H. (2021). Curcumin protection against ultraviolet-induced photo-damage in Hacat cells by regulating nuclear factor erythroid 2-related factor 2. Bioengineered.

[78] Kobayashi T., Matsumoto M., Iizuka H., Suzuki K., Taniguchi N. (1991). Superoxide dismutase in psoriasis, squamous cell carcinoma and basal cell epithelioma: An immunohistochemical study. Br. J. Dermatol..

[79] Kobayashi T., Saito N., Takemori N., Iizuka S., Suzuki K., Taniguchi N., Iizuka H. (1993). Ultrastructural localization of superoxide dismutase in human skin. Acta Derm. Venereol..

[80] Sasaki H., Akamatsu H., Horio T. (2000). Protective role of copper, zinc superoxide dismutase against UVB-induced injury of the human keratinocyte cell line HaCaT. J. Investig. Dermatol..

[81] Lyons N.M., O’Brien N.M. (2002). Modulatory effects of an algal extract containing astaxanthin on UVA-irradiated cells in culture. J. Dermatol. Sci..

[82] Ansary T.M., Hossain M.R., Kamiya K., Komine M., Ohtsuki M. (2021). Inflammatory molecules associated with ultraviolet radiation-mediated skin aging. Int. J. Mol. Sci..

[83] Williams J.D., Bermudez Y., Park S.L., Stratton S.P., Uchida K., Hurst C.A., Wondrak G.T. (2014). Malondialdehyde-derived epitopes in human skin result from acute exposure to solar UV and occur in nonmelanoma skin cancer tissue. J. Photochem. Photobiol. B Biol..

[84] Çavuşoğlu K., Kalefetoğlu Macar T., Macar O., Çavuşoğlu D., Yalçın E. (2022). Comparative investigation of toxicity induced by UV-A and UV-C radiation using allium test. Environ. Sci. Pollut. Res..

[85] Yakaew S., Itsarasook K., Ngoenkam J., Jessadayannamaetha A., Viyoch J., Ungsurungsie M. (2016). Ethanol extract of *Terminalia chebula* fruit protects against UVB-induced skin damage. Pharm. Biol..

[86] Faikrua A., Jeenapongsa R., Sila-Asna M., Viyoch J. (2009). Properties of β-glycerol phosphate/collagen/chitosan blend scaffolds for application in skin tissue engineering. ScienceAsia.

[87] Inpanya P., Faikrua A., Ounaroon A., Sittichokechaiwut A., Viyoch J. (2012). Effects of the blended fibroin/aloe gel film on wound healing in streptozotocin-induced diabetic rats. Biomed. Mater..

[88] Zhang L., Liang S., Zhang Z., Wang K., Cao J., Yao M., Qin L., Qu C., Miao J. (2023). Protective Effects of ζ-Carotene-like Compounds against Acute UVB-Induced Skin Damage. Int. J. Mol. Sci..

[89] Martin P. (1997). Wound healing--aiming for perfect skin regeneration. Science.

[90] Shaw T.J., Martin P. (2009). Wound repair at a glance. J. Cell Sci..

[91] Singer A.J., Clark R.A. (1999). Cutaneous wound healing. N. Engl. J. Med..

[92] Willenborg S., Knipper J., Ranjan R., Krieg T., Eming S. (2010). Chronic wounds and inflammation. Adv. Wound Care.

[93] Schultz G.S., Davidson J.M., Kirsner R.S., Bornstein P., Herman I.M. (2011). Dynamic reciprocity in the wound microenvironment. Wound Repair Regen..

[94] Sepp K.J., Auld V.J. (2003). RhoA and Rac1 GTPases mediate the dynamic rearrangement of actin in peripheral glia. Development.

[95] Enciso J.M., Konecny C.M., Karpen H.E., Hirschi K.K. (2010). Endothelial cell migration during murine yolk sac vascular remodeling occurs by means of a Rac1 and FAK activation pathway in vivo. Dev. Dyn..

[96] Xu Y., Zhang J., Jiang W., Zhang S. (2015). Astaxanthin induces angiogenesis through Wnt/β-catenin signaling pathway. Phytomedicine.

[97] Hodge R.G., Ridley A.J. (2016). Regulating Rho GTPases and their regulators. Nat. Rev. Mol. Cell Biol..

