# Supplementary material for: Extremophilic Solutions: The Role of Deinoxanthin in Counteracting UV-Induced Skin Harm
Source: Curr Issues Mol Biol. 2023 Oct 16;45(10):8372–94. doi: 10.3390/cimb45100528 (PMC10605247; doi:10.3390/cimb45100528)
Supplement: Supplementary file 1 [file cimb-45-00528-s001.zip › cimb-2658018-supplementary.pdf]

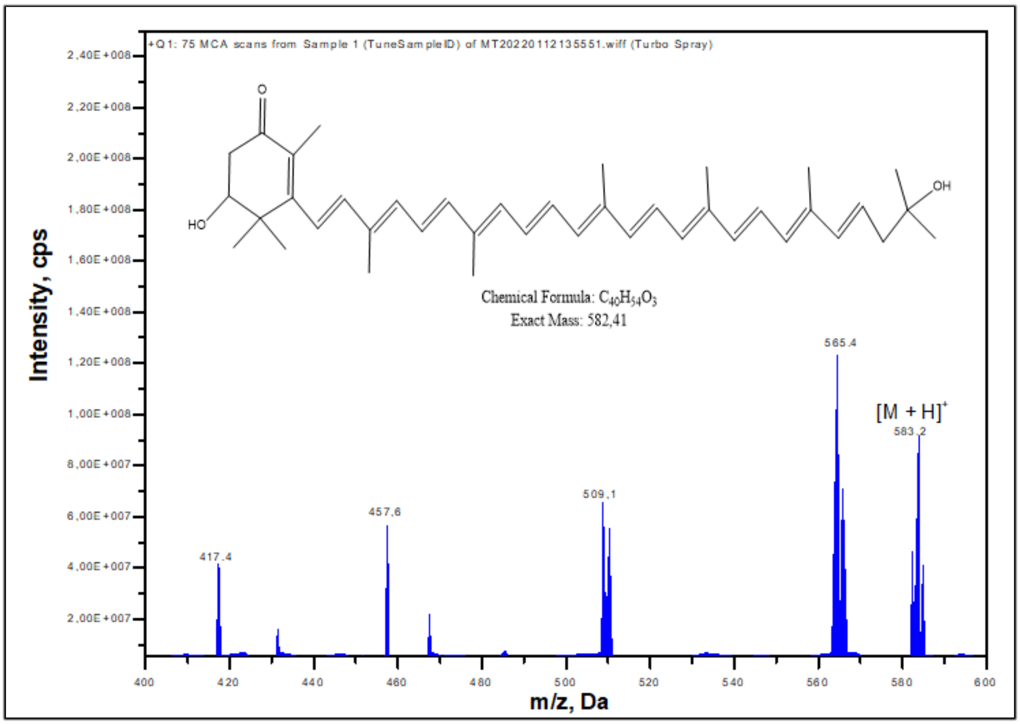

Figure S1. The isolated compound was prepared at a concentration of 50 ppb and analyzed by tandem mass spectroscopy in Q1 positive scanning mode. Ion spray voltage was set to +4500 V, declustering potential (DP) was 125.0 V, entrance potential (EP) was 10.0 V. Infusion flow rate was 10uL/min. Scanning was performed in the range of 400-600 m/z.
